# Immunogenicity and Efficacy of Monovalent and Bivalent Formulations of a Virus-Like Particle Vaccine against SARS-CoV-2

**DOI:** 10.3390/vaccines10121997

**Published:** 2022-11-24

**Authors:** Matthew D. Resch, Ke Wen, Ryan Mazboudi, Hannah Mulhall Maasz, Mirjana Persaud, Kaitlyn Garvey, Leslie Gallardo, Paul Gottlieb, Aleksandra Alimova, Reza Khayat, Jorge Morales, Helle Bielefeldt-Ohmann, Richard A. Bowen, Jose M. Galarza

**Affiliations:** 1 TechnoVax, Inc., 6 Westchester Plaza, Elmsford, NY 10523, USA; 2 CUNY School of Medicine, The City College of New York, New York, NY 10031, USA; 3 Department of Chemistry and Biochemistry, The City College of New York, New York, NY 10031, USA; 4 Microscopy Facility, Division of Science, The City College of New York, New York, NY 10031, USA; 5 School of Chemistry and Molecular Biosciences, University of Queensland, St. Lucia, QLD 4072, Australia; 6 Department of Biomedical Sciences, Colorado State University, Fort Collins, CO 80521, USA

**Keywords:** SARS-CoV-2, virus-like particle, VLP, vaccine, protective efficacy

## Abstract

Virus-like particles (VLPs) offer great potential as a safe and effective vaccine platform against SARS-CoV-2, the causative agent of COVID-19. Here, we show that SARS-CoV-2 VLPs can be generated by expression of the four viral structural proteins in a mammalian expression system. Immunization of mice with a monovalent VLP vaccine elicited a potent humoral response, showing neutralizing activity against multiple variants of SARS-CoV-2. Subsequent immunogenicity and efficacy studies were performed in the Golden Syrian hamster model, which closely resembles the pathology and progression of COVID-19 in humans. Hamsters immunized with a bivalent VLP vaccine were significantly protected from infection with the Beta or Delta variant of SARS-CoV-2. Vaccinated hamsters showed reduced viral load, shedding, replication, and pathology in the respiratory tract. Immunized hamsters also showed variable levels of cross-neutralizing activity against the Omicron variant. Overall, the VLP vaccine elicited robust protective efficacy against SARS-CoV-2. These promising results warrant further study of multivalent VLP vaccines in Phase I clinical trials in humans.

## 1. Introduction

The ongoing COVID-19 pandemic, caused by SARS-CoV-2, has resulted in more than 625 million confirmed cases and 6.5 million deaths globally [1]. Several prophylactic vaccines have received Emergency Use Listing from the World Health Organization (WHO), many of which are authorized and widely available in multiple countries [2,3]. More than 12.6 billion doses have been administered worldwide, with 65% of the population considered fully vaccinated [4]. These vaccines have proven to be successful at preventing severe disease, hospitalization, and death [5]. However, early generation vaccines are relatively ineffective at preventing infection and transmission. Even in countries with high vaccination rates, the more recent variants of SARS-CoV-2 have been able to spread rapidly [6]. Thus, a new generation of vaccines that induce a more broadly protective and longer lasting immune response is sorely needed.

SARS-CoV-2 is an enveloped positive-sense RNA virus containing four structural proteins: spike (S), membrane (M), and envelope (E) are membrane bound proteins that surround the ribonucleoprotein complex, composed of nucleocapsid (N) protein bound to viral genomic RNA [7]. The S protein, which binds to host cell receptors and facilitates viral entry [7], is the most immunodominant protein and is the primary target for neutralizing antibodies [8]. While vaccine development efforts are often focused on eliciting high titers of neutralizing antibodies, inducing a balanced humoral and cell-mediated immune response is important in reducing disease severity [9]. This is especially important given that the most recent variant of concern, Omicron, possesses several mutations in the S protein, most of which are located in the receptor binding domain [10]. These mutations allow the Omicron variant to evade neutralizing antibodies elicited by vaccination or previous infection with an earlier variant [10,11]. However, relative to the original Wuhan-Hu-1 strain, the vast majority of T-cell epitopes are conserved in the Omicron variant [12]. T-cell responses play an important role in viral clearance [13], and may even contribute to preventing initial infection [14]. T-cell epitopes are present on all proteins encoded by the genome, though S, M, and N are the three most immunodominant antigens able to stimulate CD4^+^ and CD8^+^ T-cell activity [15]. Currently approved vaccines against SARS-CoV-2 utilize different platforms, including mRNA, protein subunit, adenovirus-vector, and inactivated whole virus [2,3]. However, with the exception of inactivated whole virus, each of the vaccines based on these platforms use only the S protein as an immunogen. Next generation vaccines against SARS-CoV-2 would benefit from the inclusion of additional antigens.

Virus-like particles (VLPs) are an attractive alternative strategy for vaccine development. VLPs are self-assembled structural mimics of viruses which lack viral genomic material and are therefore noninfectious. When used as a vaccine platform, VLPs have demonstrated great safety and efficacy profiles against human papillomavirus, hepatitis B, and hepatitis E [16,17,18,19,20], due to their ability to stimulate both humoral and cellular immune responses [21,22]. Coronavirus VLP assembly and release requires expression of both the E and M proteins at a minimum [23], though S and N can also be incorporated into the particle when co-expressed [24,25]. The incorporation of all four structural proteins into VLPs provides an advantage over most current vaccine strategies against SARS-CoV-2, and does not require the chemical inactivation process necessary to produce inactivated whole virus vaccines, which can alter antigenic properties and reduce immunogenicity [26,27,28].

In this study, we generated monovalent and bivalent SARS-CoV-2 VLP vaccines composed of all four structural proteins. VLPs were produced by recombinant expression of S, E, M, and N in a mammalian expression system. Initial immunogenicity studies were conducted in mice using the monovalent VLP vaccine, produced using the Beta variant S protein. Subsequent studies were performed by immunizing Syrian Golden hamsters with a bivalent VLP vaccine, produced by combining monovalent VLPs containing either the Beta or Delta variant S protein on their surface. At the time the study was conducted, Beta and Delta were the most prevalent circulating variants of concern. Additionally, there is considerable antigenic distance between the Beta and Delta variants [29,30], and convalescent sera from patients infected with Beta is typically poor at cross-neutralizing Delta, and vice versa [29,31]. Immunization with the bivalent VLP vaccine conferred significant protection against viral challenge.

## 2. Materials and Methods

### 2.1. Ethics Statement

Mice and hamsters were used according to protocols approved by the Institutional Animal Care and Use Committees of New York Medical College and Colorado State University, respectively. Convalescent sera collected from humans (HCS) following infection with SARS-CoV-2 were obtained from BEI Resources (NIAID, NIH). HCS was deposited to BEI Resources by the Ellebedy lab prior to June of 2020, described previously [32].

### 2.2. Cell Lines and Culture Conditions

HEK-293 cells (Gibco) were grown in suspension using EX-CELL^®^ CD HEK293 viral vector medium (Millipore Sigma). 293T cells (ATCC, CRL-3216) were maintained in Dulbecco’s Modified Eagles Medium (DMEM) (ATCC, 30-2002) supplemented with 10% heat-inactivated fetal bovine serum (FBS), 2 mM L-Glutamine and 1× penicillin/streptomycin (100 U penicillin and 100 µg streptomycin per mL). A549-ACE2-TMPRSS2 cells (InvivoGen) were cultured in DMEM (Gibco, 11995065) supplemented with 10% heat-inactivated FBS and 1× penicillin/streptomycin. For maintenance of stably transfected *ACE2* and *TMPRSS2* genes, 0.5 µg/mL Puromycin and 300 µg/mL Hygromycin were added as selection antibiotics. All cultures were incubated at 37 °C with 5% CO_2_. Suspension cultures were incubated while shaking at 125 rpm.

### 2.3. Plasmid Construction

The genes encoding the 4 structural proteins of SARS-CoV-2 (spike (S), envelope (E), membrane (M), and nucleocapsid (N)) were chemically synthesized using a codon-optimized sequence and cloned into the cloning vector pUC57 by Synbio Technologies. The sequence of either the Beta (B.1.351, Genbank ON647459.1) or Delta (B.1.617.2, Genbank MZ571142.1) variant S protein was used for VLP formation as indicated. Both S protein variants were synthesized with mutations in the Furin cleavage site (Beta: RRAR_679–682_SGSA, Delta: RRAR_682–685_SGSA) and in the S2 subunit (Beta: KV_983–984_PP, Delta: KV_986–987_PP). Wild type Wuhan-Hu-1 strain sequences were used for E, M, and N (Genbank: MN908947.3), as these proteins share a high degree of sequence homology across the Wuhan-Hu-1, Beta, and Delta variants. An additional 30–40 nucleotides were synthesized at each end of the coding region of each gene, complementary to the target region within the desired expression vector. Synthesized sequences were recovered from the cloning vectors and sub-cloned by Gibson assembly to generate the engineered mammalian expression plasmid as follows: pCMV-E-SV40-N-CMV-S-SV40-M (see plasmid map, Figure S1). The assembled plasmid was transformed into MAX Efficiency Stbl2 competent cells (Life Technologies) and a correct clone was identified by restriction enzyme digestion and verified by DNA sequencing.

### 2.4. Production and Purification of SARS-CoV-2 VLPs

VLPs were produced by transient transfection of HEK293 cells with the expression plasmid pCMV-E-SV40-N-CMV-S-SV40-M using polyethylenimine (PEI Max, Polysciences, Warrington, PA, USA, 24765). Cells were seeded at 1.8 × 10^6^ cells/mL one day prior to transfection. Plasmid DNA was mixed with PEI in a 1:4 ratio (DNA:PEI) in fresh culture medium equal to 5% of the total culture volume. Mixtures were incubated at room temperature for 20 min to allow DNA/PEI complexes to form, and then added to cells dropwise. At 24 h post transfection, valproic acid was added to the culture at a final concentration of 3.75 mM. At 72 h post transfection, cultures were treated with 25 U/mL benzonase endonuclease (Millipore Sigma, Burlington, MA, USA, 1.03773) for 1 h at 37 °C to digest extracellular DNA. Cells and large debris were then clarified by filtration using Clarisolve 40 MS filters. Clarified supernatant was supplemented with Halt™ protease and phosphatase inhibitors (Thermo Scientific, Waltham, MA, USA) and 1× penicillin/streptomycin. Supernatant was concentrated 10× and buffer exchanged into binding buffer (25 mM Tris, 100 mM NaCl, pH 7.22) by tangential flow filtration using a Pellicon^®^ 2 mini 300 kDa cassette (Millipore Sigma). Concentrated sample was loaded onto a column packed with Eshmuno Q resin (Millipore Sigma) equilibrated with binding buffer. Unbound protein was removed by washing with 3 column volumes (CV) of binding buffer. Bound protein was eluted with 2 CV of 0–50% gradient elution buffer (25 mM Tris, 1 M NaCl, pH 7.22). Eluted fractions containing purified VLPs were concentrated and buffer exchanged into PBS with 10% sucrose, 30 mM L-Arginine, 20 mM L-Histidine, and 1% glycerol. Material was concentrated another 10× using 100 kDa Amicon spin filters. Purified VLPs were flash frozen and stored at −80 °C.

### 2.5. Western Blot Analysis and Densitometry

Purified VLP samples were mixed with lithium dodecyl sulfate (LDS) sample buffer (1× final concentration) and heated at 100 °C for 5 min. Samples were loaded onto 4–12% Bis-Tris gels (Invitrogen, Waltham, MA, USA) and transferred to a nitrocellulose membrane. Membranes were blocked with 5% non-fat dry milk in TBS-T (20 mM Tris, 150 mM NaCl, 0.1% Tween-20). Primary antibodies were added to membranes and incubated overnight: anti-N (1:1000 dilution, Sino Biological, Beijing China, 40143-T62), anti-M (antibody cocktail: i. (1:250 dilution, ProSci, Fort Collins, CO, USA, 3529) and ii. (1:250 dilution, Invitrogen, PA1-41160)), or anti-S (1:1000 dilution, generated in house via immunization of mice with purified S protein (BEI Resources, NIAID, Manassas, VA, USA, NR-52308)). Membranes were washed 3 times with TBS-T and subsequently incubated for 1 h with HRP-conjugated secondary antibody: anti-rabbit (1:6000 dilution, Invitrogen, 65-6120) or anti-mouse (1:6000 dilution, Invitrogen, 62-6520). Blots were developed with SuperSignal West Pico PLUS chemiluminescent substrate (Thermo Scientific) and imaged using an Azure Biosystems C600 Imaging System. Densitometry analysis was performed using AzureSpot software (Azure Biosystems, Dublin, CA, USA). Purified recombinant S protein was used as a standard (BEI Resources, NR-52308).

### 2.6. Electron Microscopy

Negative staining electron microscopy was performed by loading samples (5 μL) of purified SARS-CoV-2 VLPs onto CF200-CU carbon film 200 mesh copper grids (Electron Microscopy Sciences, Hatfield, PA, USA). Grids were incubated at room temperature for 1 min and subsequently washed with 50 mM sodium cacodylate buffer and then immediately stained with 2% phosphotungstic acid for 1 min. The grids were examined using a JOEL 2100 transmission electron microscope operating at 200 kV with an Orius 2048 × 2048-pixel CCD (Gatan Inc., Pleasanton, CA, USA). Immunogold labeling was performed by applying purified VLP samples (5 μL) onto a Formvar-coated grid followed by incubation at room temperature for 5 min. The grid was then washed 5 times with buffer (PBS with 0.1% fetal bovine serum, 10 mM glycine, 0.01% NaN_3_), fixed with 4% paraformaldehyde in PBS for 15 min, and blocked in 1% bovine serum albumin in PBS for 30 min. The grid was placed onto 40 μL of a polyclonal anti-Spike antibody (generated in house) at 1:1000 dilution in PBS with 0.1% BSA, 0.01% NaN_3_ buffer overnight at 4 °C in a humid chamber to prevent evaporation. After 5 washes with buffer, the grid was placed on a drop of goat anti-mouse IgG conjugated with 6 nm gold beads (Electron Microscopy Sciences, 25124) at a 1:3 dilution in PBS with 0.1% BSA, 0.01% NaN_3_ for 2 h at room temperature. The sample was then washed 5 times in washing buffer and stained with 2% phosphotungstic acid (pH 7.0) and examined by electron microscopy as described above.

### 2.7. Immunization of Mice

Purified SARS-CoV-2 VLPs were used to immunize 6–8-week-old BALB/c mice from Charles River Laboratories. Mice were immunized with monovalent Beta variant VLP vaccine compositions formulated with adjuvant or buffer as follows: i. aluminum hydroxide (alum), ii. AddaVax (InvivoGen, San Diego, CA, USA), or iii. non-adjuvanted VLPs (*n* = 10/group, 5 male and 5 female). Mice were primed and boosted 3 weeks later. Each dose contained 4 µg of Spike protein (as determined by densitometry of Western Blots) and was delivered via intramuscular injection. Sera were collected 2 weeks after the booster immunization.

### 2.8. Detection of S-Binding Antibodies and Antibody Subclass Analysis by ELISA

Plates were coated with purified S protein (variant-specific S protein used as indicated in respective figure legends; Wuhan (SinoBiological, 40589-V08B1), Beta (BEI Resources, NR-55310), Delta (BEI Resources, NR-55614), each used at 50 ng/well) in 0.1 M Sodium Bicarbonate Buffer (pH 9.6) and incubated overnight at 4 °C. Plates were then blocked with 1% BSA in PBS for 1 h at room temperature, and washed with 0.05% Tween 20 in TBS. Serum samples (*n* ≥ 4/group) were serially diluted 1:4 in blocking buffer and incubated for 2 h at room temperature. Plates were again washed and incubated with the following HRP-labeled secondary antibodies: for quantification of total IgG, either goat anti-mouse (1:2000 dilution, IgG heavy and light chain, Invitrogen), goat anti-hamster (1:3000 dilution, IgG heavy and light chain, SouthernBiotech), or mouse anti-human (1:2000, IgG heavy and light chain, Invitrogen); for isotype subclass analysis, goat anti-mouse IgG1 or goat anti-mouse IgG2a (1:4000 dilution, IgG1 or IgG2a heavy chain, SouthernBiotech). Secondary antibodies were diluted in blocking buffer and added to each well for 1 h at room temperature. The plates were washed for a final time and developed with TMB substrate (Thermo Scientific) for 15 min at room temperature. The reaction was stopped with 2 M sulfuric acid and the absorbance was read at 450 nm. For each serum sample, absorbance values were plotted against the serum dilution factor. A sigmoidal 4-parameter logistic regression was fitted and used to calculate the binding titer (defined as the serum dilution at which absorbance readings are at 50% of their maximum value). Binding titers that fell below the lower limit of detection were assigned a value equal to half of the starting serum dilution to allow for calculation and statistical analysis.

### 2.9. Pseudotyped Lentivirus Neutralization Assay

Sera from immunized animals were analyzed for the presence of neutralizing antibodies using a pseudovirus neutralization assay as described previously [33]. Briefly, replication-incompetent lentiviruses pseudotyped with the SARS-CoV-2 spike protein were produced by transfecting 293T cells using a lentiviral plasmid kit (BEI Resources, NR53816) with two modifications. Instead of using the Wuhan-Hu-1 S protein (BEI Resources, NR-53742), we used a plasmid encoding either the Beta (B.1.351), Delta (B.1.617.2), or Omicron (B.1.1.529, BA.1 subvariant, Genbank OP057208.1) variant S protein. Additionally, we used a lentiviral backbone plasmid encoding only the ZsGreen reporter (BEI Resources, NR-52520), in place of the backbone plasmid included in the kit which encodes both ZsGreen and luciferase (BEI Resources, NR-52516).

Neutralization assays were performed in 96-well plates that had been coated with poly-L-lysine (Millipore Sigma, P4707) and seeded with 1.25 × 10^4^ A549-ACE2-TMPRSS2 cells/well 24 h prior to assay. Heat-inactivated sera from immunized animals (*n* ≥ 3/group) were serially diluted 2-fold in a separate 96-well plate in growth medium (DMEM, 1% FBS), in a final volume of 100 µL. Serum from each animal was run in triplicate. Each dilution was mixed with an equal volume of pseudovirus containing 300 fluorescence forming units (FFU). Polybrene (Sigma Aldrich, St. Louis, MO, USA, TR-1003-G) was added to each mixture at a final concentration of 5 µg/mL to facilitate lentiviral infection. Mixtures containing the diluted serum and pseudovirus were incubated for 1 h at 37 °C and then applied to A549-ACE2-TMPRSS2 cells following the removal of the growth medium. Plates were then allowed to incubate for 3 days at 37 °C with 5% CO_2_. The following controls were included: i. serum harvested from mice prior to immunization (Control), ii. SARS-CoV-2 convalescent serum collected from humans (HCS) prior to June of 2020 (BEI Resources), iii. cells exposed only to pseudovirus (no serum), and iv. cells that were unexposed to pseudovirus. After the incubation period, the growth medium was replaced with PBS to reduce background fluorescence. Plates were then imaged and analyzed using a Celigo Image Cytometer (Nexcelom Bioscience, Lawrence, MA, USA). The number of green fluorescent cells in each well were quantified and plotted against the serum dilution factor. For each animal, a non-linear regression was fitted and used to calculate the inhibitory dilution at which the serum neutralized 50% of the pseudovirus relative to the control wells that contained pseudovirus without any serum (ID_50_). Neutralizing antibody titers that fell below the lower limit of detection were assigned a value equal to half of the starting serum dilution to allow for calculation and statistical analysis.

### 2.10. Viruses

The two viruses used for challenge and serologic testing were obtained originally from BEI Resources and passaged once in Vero cells prior to use: i. Beta variant (B.1.351, BEI NR-54008), and ii. Delta variant (B.1.617.2, BEI NR-55612).

### 2.11. Viral Challenge Study in Golden Syrian Hamsters

A bivalent VLP vaccine adjuvanted with AddaVax was used to immunize 9–10-week-old Golden Syrian hamsters from Charles River Laboratories. The bivalent VLP vaccine was generated by combining equal amounts (as determined by densitometry of anti-S Western blot) of purified monovalent VLPs produced with either the Beta or Delta variant S protein. Hamsters were acclimated for 7 days before immunization with either a low- (5 µg total Spike) or high- (10 µg total Spike) dose of bivalent VLPs, or control (PBS) (*n* = 24 per group, 12 males and 12 females). Booster immunization was administered 2 weeks after the first dose. Each immunization was administered by intramuscular injection into a rear leg. Blood samples were collected 3 weeks after booster immunization and sera were stored frozen. After collection of sera, animals were challenged by intranasal instillation of 100 µL containing 10^4^ plaque-forming units (PFU) of either the Beta or Delta variant of SARS-CoV-2 under ketamine-xylazine anesthesia (*n* = 12 per group per challenge variant). Animals were observed and weighed daily beginning at the time of challenge and extending to the day of euthanasia. On days 1, 2, and 3 post challenge, oropharyngeal swab samples were collected from all hamsters into viral transport medium (tris-buffered minimal essential medium containing 1% BSA, 5% FBS, gentamicin, penicillin, and streptomycin) and stored frozen until assay. Half of the hamsters in each vaccine group were euthanized 3 days post challenge and samples of nasal turbinates, right cranial and right caudal lung lobes were homogenized and stored frozen for virus assay/RNA extraction. The remainder of the lung was fixed by immersion in formalin. The remaining hamsters in each group were euthanized 7 days post challenge and tissue samples were collected and stored as described above.

### 2.12. Plaque Assay and Plaque Reduction Neutralization Titers (PRNT)

Titers of stock viruses and swab/tissue samples from hamsters (*n* ≥ 5/group) were determined using a double overlay plaque assay on Vero cells as previously described [34]. Titers that fell below the lower limit of detection (10 PFU/swab or 10 PFU/100 mg tissue) were assigned a value of 5 PFU to allow for calculation and statistical analysis.

Sera collected on the day of challenge were tested for neutralizing antibodies by plaque reduction neutralization assay. Briefly, serial dilutions of sera (1:10–1:320) were mixed with an equal volume of either the Beta or Delta variant of SARS-CoV-2. Mixtures were incubated for 60 min at 37 °C, then inoculated onto Vero cell monolayers in 6-well plates. Plaques in each well were quantified and used to determine the neutralizing antibody titers, expressed as the maximum dilution of serum that reduced plaque counts by >50% (PRNT_50_) relative to cells incubated with virus in the absence of serum. PRNT_50_ values that fell below the lower limit of detection were assigned a value equal to half of the starting serum dilution to allow for calculation and statistical analysis. PRNT_50_ values that were above the upper limit of detection were assigned a value of 320, equal to the maximum serum dilution tested.

### 2.13. Histopathology

Lung tissues collected from hamsters on day 3 or 7 post challenge were fixed by immersion in buffered formalin and processed to generate slides stained with hematoxylin-eosin. Slides were evaluated in a blinded manner by a veterinary pathologist. Right medial and left lung lobes were scored using a previously described scoring system [34]. Briefly, lobes were scored separately for overall lesion extent, bronchitis, alveolitis, pneumocyte hyperplasia, vasculitis, and interstitial inflammation, each on a 0–4 or 0–5 scale. The scores for each lobe were summed.

### 2.14. Extraction of Viral RNA from Infected Tissue and RT-qPCR

Viral RNA was extracted from homogenized tissue samples that were stored frozen in TRIzol reagent (Invitrogen). Tissue samples included nasal turbinates collected from animals euthanized 3 days post challenge, and right cranial lung from animals euthanized 3- and 7 days post challenge (*n* ≥ 3/group, except in day 3 cranial lung samples from control group challenged with Beta variant, where *n* = 2). Frozen samples were thawed at room temperature and RNA was extracted using the Direct-zol RNA Miniprep Plus kit (Zymo Research, R2070, Irvine, CA, USA) following the manufacturer’s protocol. RNA was eluted in DNase/RNase free water and stored at -80 °C. RT-qPCR was conducted using the TaqMan™ Fast Virus 1-Step Master Mix (Applied Biosystems, 4444432, Waltham, MA, USA). The diagnostic primer/probe set, designed by Hong Kong University [35], targeting the nsp14 region of ORF1b was used in this study to detect and amplify genomic RNA. The primer and probe sequences are: 5′-TGGGGYTTTACRGGTAACCT-3′ (Forward), 5′-AACRCGCTTAACAAAGCACTC-3′ (Reverse), and 5-TAGTTGTGATGCWATCATGACTAG-3′ (Probe, with 5′ fluorescent reporter 6-FAM and 3′ quencher BHQ-1). Each sample was run in duplicate, using 100 ng of total RNA as template for each reaction. RNA transcript standards were used for quantification of sample RNA and were produced as previously described [36]. Briefly, cDNA encoding an ~850 base region of nsp14 was synthesized from genomic RNA (BEI Resources, NR-52285, GenBank: MN985325.1) using the following primers: 5′-TAATACGACTCACTATAGGGTAGTGCTAAACCACCGCCTG-3′ (Forward, adding the T7 promoter sequence to the 5′ end of the target amplicon), and 5′-AACTGCCACCATCACAACCA-3′ (Reverse). cDNA was gel purified and transcribed in vitro using the HiScribe T7 ARCA mRNA kit (New England Biolabs, Ipswich, MA, USA, E2060S), with DNase treatment. RNA was purified using the Monarch RNA Cleanup kit (New England Biolabs, T2040S) and quantified using a NanoDrop One spectrophotometer (Thermo Scientific). Standard curves were generated using 10-fold serially diluted RNA standards. DNase/RNase free water was used as a non-template control. RT-qPCR reactions were performed using the QuantStudio 5 Real-Time PCR system (Applied Biosystems) using the following thermocycling conditions: 50 °C for 5 min (reverse transcription), 95 °C for 20 s (inactivation of reverse transcriptase), followed by 40 cycles of 95 °C for 5 s, 60 °C for 30 s (PCR amplification). RNA copy numbers that fell below the lower limit of detection were assigned a value equal to half of the lowest value used in generating the standard curve to allow for calculation and statistical analysis.

### 2.15. Statistical Analysis

Results were analyzed by one-way or two-way analysis of variance (ANOVA) with Tukey’s multiple comparisons test, as indicated. Differences were considered significant at *p* values of <0.05. Correlation coefficients were calculated using Pearson’s correlation analysis. All statistical analyses were performed using GraphPad Prism 9 software (GraphPad Software, San Diego, CA, USA).

## 3. Results

### 3.1. Production, Purification, and Characterization of SARS-CoV-2 VLPs

**Figure 1:**
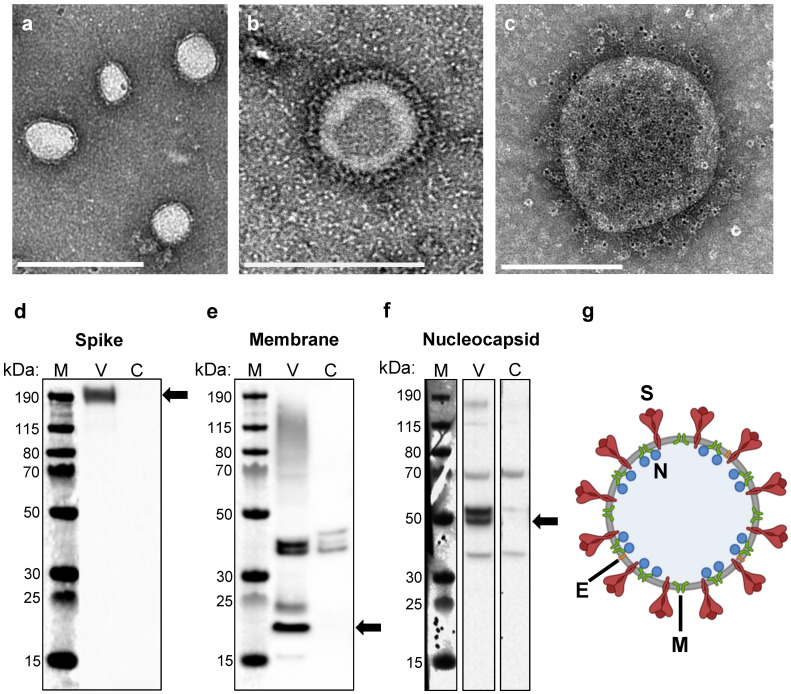
**Construction of SARS-CoV-2 VLP vaccines**. (**a**,**b**) Electron micrographs of purified SARS-CoV-2 VLPs negatively stained with phosphotungstic acid. Scale bars are 500 nm and 200 nm in (**a**) and (**b**), respectively. (**c**) Immunogold-labeled electron micrograph generated using an anti-spike primary antibody and gold-bead labeled secondary antibody. Gold beads appear as small black dots, ~6 nm in diameter. Scale bar 200 nm. (**d**–**f**) Western blot analysis of purified VLPs (V) and mock transfected cell lysate as a negative control (C) generated using an (**d**) anti-spike antibody, (**e**) anti-membrane antibody, and (**f**) anti-nucleocapsid antibody. The sizes of the molecular weight markers (M) are labeled to the left of each blot. Arrows indicate the protein of interest for each blot. Full images of each blot are shown in Figure S2. (**g**) Schematic of a SARS-CoV-2 VLP, containing all four structural proteins (S, E, M, and N) and devoid of genetic material.

SARS-CoV-2 VLPs were produced by transient transfection of HEK293 cells with a single plasmid encoding all four viral structural proteins (S, E, M, and N, plasmid schematic, Figure S1). We used an S protein sequence with mutations in the S1/S2 Furin cleavage site and in the S2 subunit between Heptad Repeat 1 and the Central Helix. These mutations have been previously shown to stabilize the S protein in its more immunogenic pre-fusion conformation [37,38,39,40]. Three days post transfection, self-assembled VLPs were collected from the culture supernatant and purified using a sequential process of filtration, tangential flow filtration/buffer exchange, and anion exchange chromatography. Formation of VLPs was confirmed and analyzed by electron microscopy. Purified VLPs exhibited a predominantly spherical morphology with diameters ranging from 100 to 140 nm, and also contained a coat of surface projections, closely resembling the native virus (Representative images, Figure 1a,b). Immunogold labeling using an anti-S antibody confirmed that these projections were composed of S protein (Figure 1c). VLP composition was analyzed by Western blot, revealing that purified particles contained the three most abundant structural proteins, S, M, and N (Figure 1d–f). We were unable to detect E by Western blot, likely due to the low abundance of E in assembled particles [41]. A schematic of a SARS-CoV-2 VLP is shown in Figure 1g.

### 3.2. Immunization with a SARS-CoV-2 VLP Vaccine Elicits Robust Production of S-Binding and Neutralizing Antibodies in Mice

**Figure 2:**
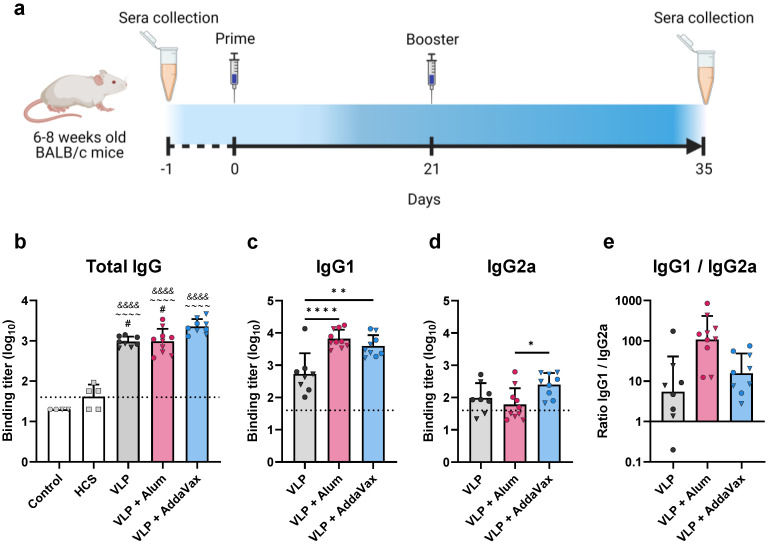
**Mouse vaccination schedule and assessment of immunogenicity via ELISA.** (**a**) Immunization schedule for mice vaccinated with the monovalent (Beta) VLP vaccine. Groups of mice (*n* ≥ 8) were immunized with the monovalent VLP vaccine alone (VLP), or adjuvanted with aluminum hydroxide (alum) or AddaVax. Prime and booster immunizations were administered on days 0 and 14, respectively. Serum samples were collected prior to immunization (day^−1^) and three weeks after the booster immunization (day 35). (**b**–**e**) Sera were assessed by ELISA for Spike-specific antibodies using plates coated with the Wuhan-Hu-1 Spike protein. Binding titer represents the dilution at which sera showed half of their maximal binding activity. Binding titers for individual animals are presented as either circles (●) for females or triangles (▼) for males. Dotted line indicates lower limit of detection. (**b**) Total IgG levels were measured for all vaccinated mice and compared to human convalescent sera (HCS) and sera collected from mice prior to immunization (Control). Spike-specific (**c**) IgG1 and (**d**) IgG2a were measured in vaccinated mice. (**e**) Ratio of IgG1 to IgG2a. Data are mean + SD in (**b**–**d**), and geometric mean + geometric SD in (**e**). Results were analyzed by one-way ANOVA with Tukey’s multiple comparisons test, with *p* < 0.05 considered significant. * *p* < 0.05, ** *p* < 0.01, **** *p* < 0.0001. In (**b**), ampersands (&) indicate comparisons with Control, tildes (~) indicate comparisons with HCS, and pound signs (#) indicate comparisons with the VLP + AddaVax group. In (**c**–**e**), statistical comparisons are represented with asterisks above lines connecting the compared groups.

Initial immunogenicity studies were conducted in a murine model using a monovalent VLP vaccine assembled with the Beta (B.1.351) variant S protein. Groups of BALB/c mice were vaccinated with purified VLPs alone, or in combination with either aluminum hydroxide (alum) or AddaVax as an adjuvant. Mice were primed and then boosted three weeks later, with each dose containing 4 µg of S protein (immunization schedule, Figure 2a). Serum samples were collected two weeks after the booster immunization and evaluated for Wuhan-Hu-1 S protein-specific IgG antibodies by ELISA. Immunization with VLPs elicited high titers of S-specific antibodies. VLP-immunized mice showed significantly higher binding titers as compared to human convalescent sera (HCS), regardless of adjuvant formulation (Figure 2b). Sera collected from mice prior to immunization did not demonstrate S protein-specific binding (Figure 2b). Immunization with VLPs adjuvanted with AddaVax induced significantly higher antibody titers compared to the alum and non-adjuvanted groups (Figure 2b). We also determined the levels of IgG1 (Figure 2c) and IgG2a (Figure 2d) elicited by vaccination, as these markers are indicative of a Th2- or Th1-biased response, respectively. IgG subclass analysis revealed that VLPs adjuvanted with alum elicited primarily IgG1, indicative of a strong Th2 response, while all other adjuvant groups demonstrated a more balanced response (Figure 2e).

**Figure 3:**
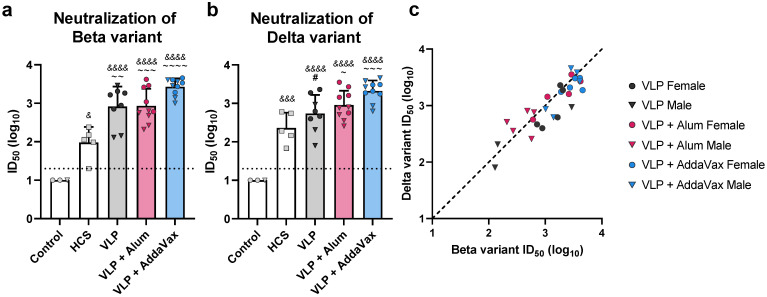
**Functional antibody response to the monovalent VLP vaccine.** Neutralizing antibody titers in sera collected from VLP-immunized mice, sera collected from mice prior to immunization (Control), and human convalescent sera (HCS) were measured using a Spike-pseudotyped lentivirus neutralization assay. (**a**) Beta variant Spike-pseudotyped lentivirus neutralization. (**b**) Delta variant Spike-pseudotyped lentivirus neutralization. Neutralizing antibody titers are presented as the serum dilution at which half of the pseudovirus was neutralized as compared to serum-negative controls (ID_50_). ID_50_ values for individual animals are presented as either circles (●) for females or triangles (▼) for males. Dotted line indicates lower limit of detection. (**c**) Correlation of neutralizing antibody titers against the Beta and Delta variants. Dashed black line indicates a slope of 1, included to visualize the skew of the data. In (**a**) and (**b**), data are mean + SD. Results were analyzed by one-way ANOVA with Tukey’s multiple comparisons test, with *p* < 0.05 considered significant. Ampersands (&) indicate comparisons with Control, tildes (~) indicate comparisons with HCS, and pound signs (#) indicate comparisons with the VLP + AddaVax group. * *p* < 0.05, ** *p* < 0.01, *** *p* < 0.001, **** *p* < 0.0001 (The asterisks in the figure legend represent all statistical comparisons, regardless of symbol used. #, &, and ~ in the figure are represented by asterisks in the figure legend, to significantly shorten the length of the legend).

Sera were further analyzed for the presence of neutralizing antibodies using a pseudotyped lentivirus neutralization assay [33]. To measure the ability of VLP immunization to elicit cross-neutralizing antibodies, neutralizing activity against both the vaccine-homologous Beta variant and -heterologous Delta variant (B.1.617.2) was determined. Immunization with VLPs elicited high titers of broadly protective neutralizing antibodies. Compared to human convalescent sera, all vaccinated groups showed 8- to 28-fold higher mean 50% neutralization titers (ID_50_) against the Beta variant (Figure 3a, mean ID_50_ values: HCS = 10^1.98^, VLP = 10^2.92^, alum = 10^2.93^, AddaVax = 10^3.43^). Against the Delta variant, VLP-immunized mice showed 2- to 9-fold higher neutralizing antibody titers relative to HCS (Figure 3b, mean ID_50_ values: HCS = 10^2.36^, VLP = 10^2.74^, alum = 10^2.96^, AddaVax = 10^3.32^). In contrast, sera collected from naïve mice did not neutralize either variant. Furthermore, mice immunized with VLPs adjuvanted with AddaVax produced the highest titers of neutralizing antibodies, demonstrating approximately 2-fold higher titers against both the Beta and Delta variant as compared to all other adjuvant groups (Figure 3a,b). We also found that within adjuvant groups, females tended to produce slightly higher titers of neutralizing antibodies than males, though these differences were not statistically significant. At the individual animal level, titers of neutralizing antibodies against the Beta variant correlated with those against the Delta variant (R^2^ = 0.78, *p* < 0.0001, Figure 3c). Titers tended to be slightly higher against the Beta variant, which was expected given that VLPs were produced using the Beta variant S protein.

### 3.3. Efficacy of a Bivalent VLP Vaccine against SARS-CoV-2 Challenge in the Golden Syrian Hamster Model

**Figure 4:**
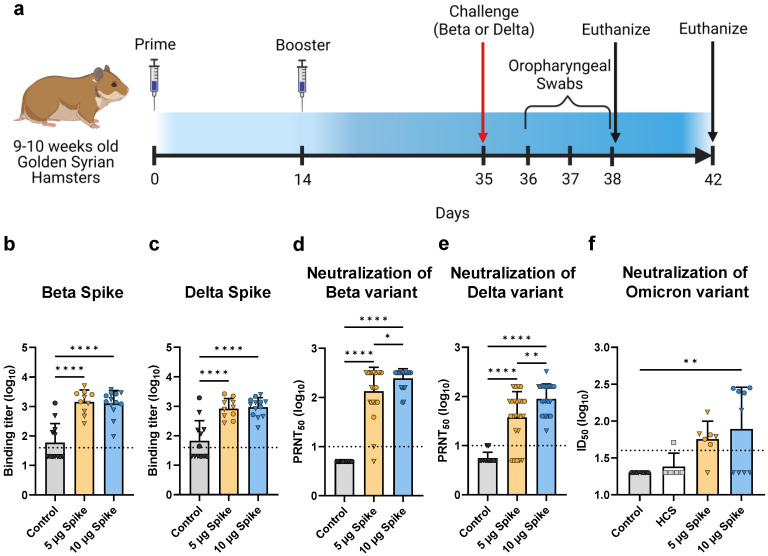
**Hamster vaccination schedule and assessment of humoral immunity in response to bivalent VLP vaccination.** (**a**) Immunization schedule for hamsters vaccinated with the bivalent (Beta and Delta) VLP vaccine adjuvanted with AddaVax. Bivalent VLPs were administered as either a low- (5 µg Spike) or high- (10 µg Spike) dose, and a third group was mock injected with only PBS (Control) (*n* = 12/group per challenge variant). Sera were collected on day 35 and hamsters were subsequently challenged with either the Beta or Delta variant of SARS-CoV-2. Half the animals in each group were euthanized on day 38, and the remaining animals on day 42. (**b**,**c**) Spike-specific total IgG titers determined by ELISA using plates coated with either the (**b**) Beta variant Spike or (**c**) Delta variant Spike. Binding titer represents the dilution at which sera showed half of their maximal binding activity. Neutralizing activity was measured against both the (**d**) Beta and (**e**) Delta variants of SARS-CoV-2 using a plaque reduction neutralization test (PRNT). Neutralizing antibody titers are presented as the highest serum dilution at which 50% of the virus was neutralized relative to serum-negative controls (PRNT_50_). (**f**) Neutralizing activity was measured against the Omicron variant using a pseudotyped lentivirus neutralization assay. Human convalescent sera (HCS) were also tested for neutralizing activity against Omicron. In (**b**–**f**), values for individual animals are presented as either circles (●) for females or triangles (▼) for males. Dotted line indicates lower limit of detection. Data are mean + SD. Results were analyzed by one-way ANOVA with Tukey’s multiple comparisons test, with *p* < 0.05 considered significant. * *p* < 0.05, ** *p* < 0.01, **** *p* < 0.0001.

To investigate the protective efficacy conferred by immunization with our VLP platform, we conducted a viral challenge study using the Golden Syrian Hamster model, which closely resembles SARS-CoV-2 infection in humans [42,43]. To broaden the antigenic composition of the vaccine, we combined two monovalent VLPs, produced using either the Beta or Delta variant S protein. Beta and Delta were selected given their antigenic distance [29,30] and prevalence as the predominant circulating variants at the time the study was conducted. The generated bivalent VLP vaccine contained an equal amount of the Beta and Delta variant VLPs. Hamsters were immunized with bivalent VLPs adjuvanted with AddaVax following a prime and boost regimen. Bivalent VLPs were administered to groups as either a low- (5 µg S protein) or high- (10 µg S protein) dose, with a third control group receiving only PBS. Three weeks after receiving the booster dose, serum samples were collected from hamsters and examined for S-specific binding and neutralizing antibodies. Immediately after sera collection, half of the hamsters in each group were challenged via intranasal instillation with 10^4^ PFU of the Beta variant of SARS-CoV-2, and the other half challenged with an equivalent dose of the Delta variant. Oropharyngeal swab samples were collected on each of the first 3 days after challenge. Subsequently, animals were euthanized at either 3- or 7 days post challenge to be evaluated for histopathological changes and to determine the viral load in the lungs and nasal turbinates. A schedule of the study is presented in Figure 4a.

### 3.4. Bivalent VLP Vaccination Elicits High Titers of S-Binding and Neutralizing Antibodies

Serum samples collected from hamsters 3 weeks after the booster immunization were assessed for S protein-specific antibodies via ELISA. Binding titers were determined for both the Beta and Delta variant S proteins (Figure 4b,c). Sera from hamsters immunized with either the low- or high-dose bivalent VLP vaccine showed significantly higher binding to both the Beta and Delta variant S proteins as compared to the control group, which demonstrated minimal reactivity. Interestingly, vaccine dosage did not have an effect on S-specific total IgG levels, as no differences in binding titers were observed between low- and high-dose bivalent VLP groups. Additionally, immunized hamsters showed comparable binding to Beta and Delta S proteins (*p* > 0.85 as determined by two-way ANOVA).

Sera were also assessed for neutralizing antibodies using a plaque reduction neutralization test using either the Beta or Delta variant of SARS-CoV-2. All vaccinated hamsters showed significantly higher titers of neutralizing antibodies against Beta (Figure 4d, mean PRNT_50_ values: 5 µg Spike = 10^2.11^, 10 µg Spike = 10^2.39^) and Delta (Figure 4e, mean PRNT_50_ values: 5 µg Spike = 10^1.58^, 10 µg Spike = 10^1.95^) as compared to control groups, which did not show neutralizing activity against either variant. Interestingly, vaccinated hamsters showed greater neutralizing activity against Beta than against Delta (*p* < 0.001 as determined by two-way ANOVA). Several animals showed PRNT_50_ values against Beta that were above the upper limit of detection, while PRNT_50_ values against Delta fell within the detectable range. Additionally, against both Beta and Delta, the high dose group showed significantly higher titers of neutralizing antibodies relative to the low dose group, demonstrating a dose-dependent enhancement of neutralization.

### 3.5. Cross-Neutralization of the Omicron Variant

There is considerable antigenic variation between the bivalent VLP vaccine components (Beta and Delta) and the predominant currently circulating variant, Omicron [44,45]. To assess the ability of the bivalent VLP vaccine to elicit cross-neutralizing antibodies, we measured neutralizing activity against the Omicron BA.1 variant using a pseudotyped lentivirus neutralization assay. Hamsters immunized with the low dose of the bivalent VLP vaccine exhibited moderate cross-neutralizing antibody titers as compared to the control group, whereas the high dose group showed significant neutralizing activity against Omicron, but only in the female subset of the group (Figure 4f). In contrast, the male counterpart of the group did not show considerable neutralizing activity. Interestingly, males and females of the high dose group showed comparable neutralizing activity against the Beta and Delta variants and showed similar protection against virus challenge (see below), suggesting that the lack of cross-neutralizing activity against Omicron in the male subset is not simply due to a weaker overall immune response.

### 3.6. Bivalent VLP Vaccination Significantly Reduces Viral Load and Viral RNA in the Upper and Lower Respiratory Tract

**Figure 5:**
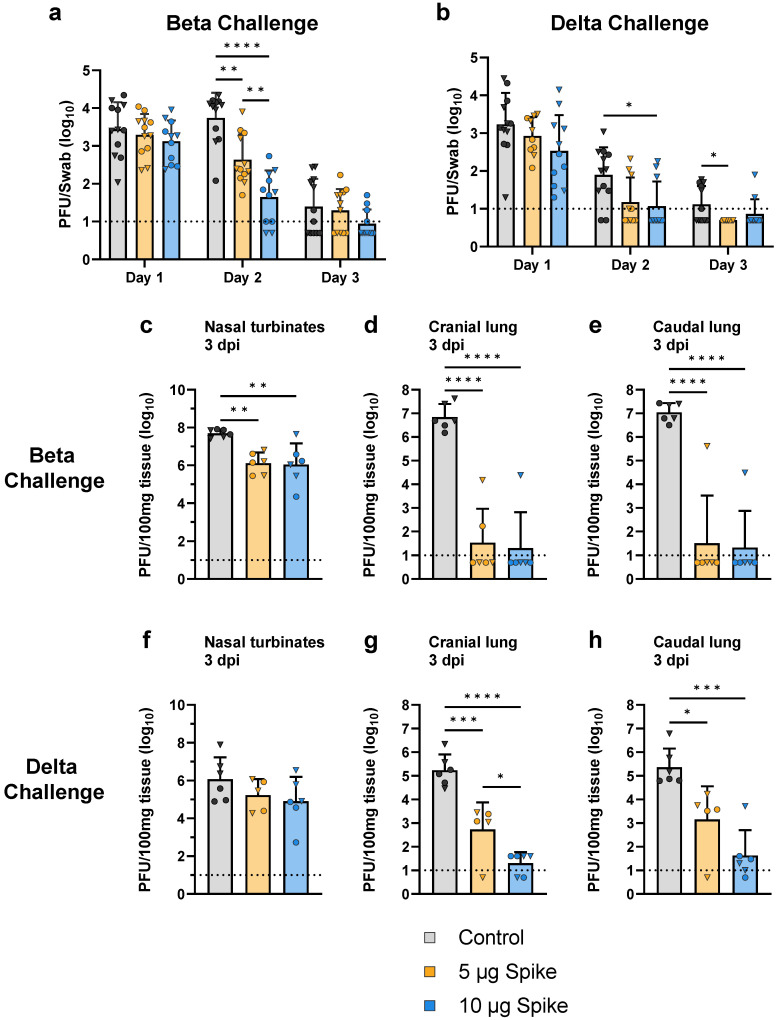
**Viral load in the upper and lower respiratory tract after challenge with SARS-CoV-2.** Viral load in the oropharyngeal passages was determined by plaque assay of swab samples collected from hamsters challenged with the (**a**) Beta or (**b**) Delta variant of SARS-CoV-2. Swab samples were collected on each of the first 3 days after challenge. Viral load was also determined by plaque assay of tissue samples collected 3 days post infection (dpi) from hamsters challenged with the (**c**–**e**) Beta or (**f**–**h**) Delta variant. Sampled tissues include the (**c**,**f**) nasal turbinates, (**d**,**g**) cranial lung, and (**e**,**h**) caudal lung. Values for individual animals are presented as either circles (●) for females or triangles (▼) for males. Dotted line indicates lower limit of detection. Data are mean + SD. Results were analyzed by two-way ANOVA with Tukey’s multiple comparisons test in (**a**) and (**b**), and by one-way ANOVA with Tukey’s multiple comparisons test in (**c**–**h**), with *p* < 0.05 considered significant. * *p* < 0.05, ** *p* < 0.01, *** *p* < 0.001, **** *p* < 0.0001.

Following the collection of sera, hamsters were challenged with either the Beta or Delta variant of SARS-CoV-2. To evaluate the protective efficacy provided by the bivalent VLP vaccine, viral load and genomic RNA were measured in the upper and lower respiratory tract at multiple timepoints after challenge. In the oropharyngeal passages, viral load was determined by plaque assay of swab samples collected on days 1, 2, and 3 post challenge. When challenged with either the Beta or Delta variant of SARS-CoV-2, vaccinated hamsters showed a reduction in viral load in the oropharynx as compared to the control group (Figure 5a,b). On day 1 post challenge, vaccinated animals demonstrated slightly lower oropharyngeal swab titers as compared to controls regardless of challenge variant, though these differences were not statistically significant. The most significant differences were observed on day 2, when low- and high-dose vaccinated animals challenged with the Beta variant showed a 12- and 120-fold reduction in viral load in the oropharynx, respectively, relative to control (Figure 5a). A similar trend was observed on day 2 in animals challenged with the Delta variant, as vaccinated hamsters showed viral titers in the oropharynx that were below the lower limit of detection, while the control group still showed a detectable amount of virus (Figure 5b). By day 3, oropharyngeal swab titers for all animals were near or below the lower limit of detection, even in the control groups.

On day 3 post challenge, half of the animals in each group were euthanized to assess viral replication by measuring viral load in the nasal turbinates as well as the cranial and caudal lobes of the lungs. Vaccination with both the low- and high-dose bivalent VLP vaccine provided robust protection against viral replication (Figure 5c–h). Compared to the control group, both low- and high-dose VLP-immunized hamsters showed a significantly lower viral load in the nasal turbinates (~40-fold reduction) and cranial and caudal lobes of the lungs (~10^5^-fold reduction) when challenged with the Beta variant (Figure 5c–e). Hamsters challenged with the Delta variant showed significantly lower titers in both the cranial (3.2 × 10^2^- and 8.6 × 10^3^-fold reduction for low- and high-dose, respectively) and caudal (1.6 × 10^2^- and 5.4 × 10^3^-fold reduction for low- and high-dose, respectively) lobes of the lungs relative to control, and a notable (~10-fold) but not statistically significant reduction in the nasal turbinates (Figure 5f–h).

**Figure 6:**
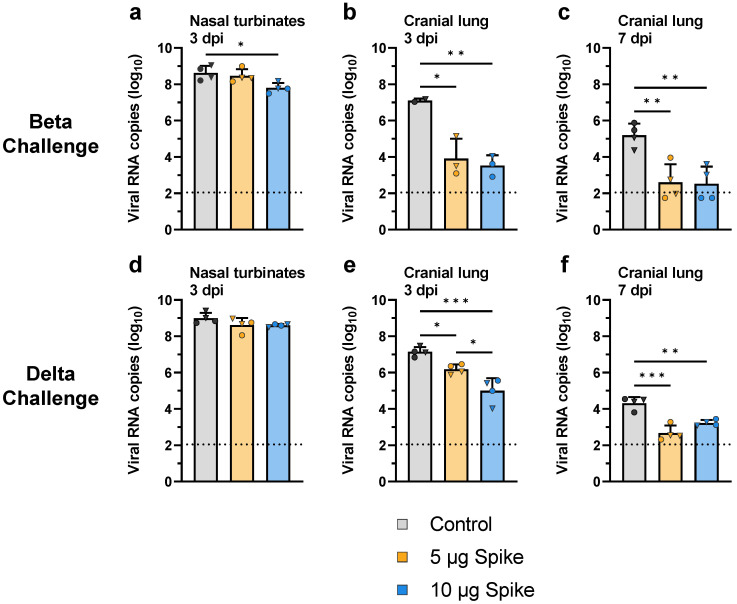
**Viral RNA in the upper and lower respiratory tract after challenge with SARS-CoV-2.** Viral RNA was quantified by RT-qPCR of RNA extracted from tissue samples collected from hamsters challenged with the (**a**–**c**) Beta or (**d**–**f**) Delta variant of SARS-CoV-2. Sampled tissues include (**a**,**d**) nasal turbinates collected 3 days post challenge, as well as cranial lung samples collected on (**b**,**e**) day 3 and (**c**,**f**) day 7 post challenge. Values indicate viral RNA copies per 100 ng of extracted RNA. Values for individual animals are presented as either circles (●) for females or triangles (▼) for males. Dotted line indicates lower limit of detection. Data are mean + SD. Results were analyzed by one-way ANOVA with Tukey’s multiple comparisons test, with *p* < 0.05 considered significant. * *p* < 0.05, ** *p* < 0.01, *** *p* < 0.001.

Protection was further evaluated by measuring viral genomic RNA in tissue samples from the nasal turbinates (day 3) and cranial lung (days 3 and 7 post challenge) using RT-qPCR. When challenged with the Beta variant, hamsters immunized with the high dose bivalent VLP vaccine showed a significant 7.5-fold reduction in viral RNA in the nasal turbinates as compared to control (Figure 6a). In the cranial lung, both low- and high-dose VLP groups showed significantly less viral RNA relative to control 3 days after challenge with the Beta variant (1.3 × 10^2^- and 1.2 × 10^3^-fold reduced, respectively), and by day 7 viral RNA was near or below the lower limit of detection in vaccinated animals, while control hamsters still showed a substantial amount of viral RNA (Figure 6b,c). Similar results were seen in animals challenged with the Delta variant. Although no difference in viral RNA copy number was observed in the nasal turbinates between vaccinated and control groups challenged with Delta (Figure 6d), low- and high-dose VLP immunized hamsters showed significantly lower amounts of viral RNA relative to control in the cranial lung (Figure 6e,f). On day 3, a dose-dependent reduction in viral RNA in the cranial lung was observed, as low-dose VLP immunized animals showed 9-fold less viral RNA as compared to control, while the high-dose group showed an ~85-fold reduction (Figure 6e). On day 7, viral RNA was near the lower limit of detection in vaccinated hamsters, while control animals showed significantly more RNA (Figure 6f), similar to the results seen in the Beta challenge group. These results parallel the infectious viral titers as determined by plaque assay (Figure 5c–h).

### 3.7. Bivalent VLP Vaccination Provides Protection against Pathology

**Figure 7:**
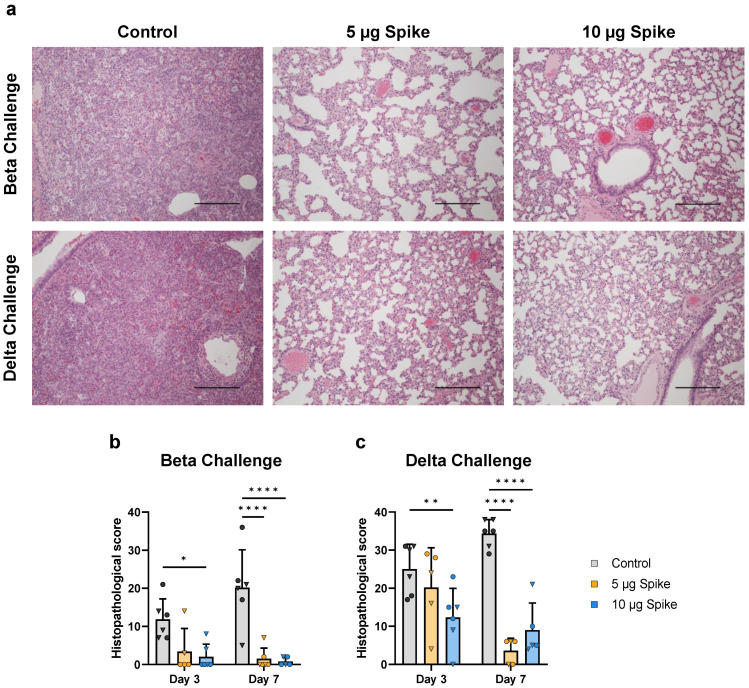
**Lung histopathology at 3- and 7 days post challenge.** (**a**) Representative micrographs of lungs from control and vaccinated animals 7 days post challenge with either the Beta or Delta variant of SARS-CoV-2. Lungs of the unvaccinated (Control) animals are characterized by loss of normal lung architecture due to severe leukocyte infiltration in alveolar spaces and septae, presence of fibrin strands in alveoli, enlargement (hypertrophy) and proliferation (hyperplasia) of pneumocytes and variable hemorrhage in the parenchyma, resulting in loss of patent airspaces (left panels). In contrast, lungs of the vaccinated animals have no or minimal leukocyte infiltration regardless of vaccine dose and alveoli are patent (middle and right panels). Scale bars are 400 µm. Histopathological scores were determined for hamsters challenged with the (**b**) Beta or (**c**) Delta variant. Scores for individual animals are presented as either circles (●) for females or triangles (▼) for males. Dotted line indicates lower limit of detection. Data are mean + SD. Results were analyzed by two-way ANOVA with Tukey’s multiple comparisons test, with *p* < 0.05 considered significant. * *p* < 0.05, ** *p* < 0.01, **** *p* < 0.0001.

To assess the effect of vaccination on disease severity, hamsters euthanized on days 3 and 7 post challenge were evaluated by histological examination of right medial and left lung lobes via H&E staining and scoring. Vaccination with bivalent VLPs provided significant protection against SARS-CoV-2-induced severe lung pathology (representative images, Figure 7a). At 3 days post challenge with either the Beta or Delta variant, hamsters vaccinated with the high dose bivalent VLP vaccine showed significantly lower histopathological scores as compared to control (Figure 7b,c). On day 7, both the low- and high-dose bivalent VLP-immunized hamsters showed minimal signs of tissue damage, regardless of challenge virus variant, while both control groups showed signs of severe disease (Figure 7b,c). This observed protection against lung pathology indicates the capability of the VLP platform to protect against SARS-CoV-2-induced severe disease.

## 4. Discussion

Several vaccines against SARS-CoV-2 have been developed and authorized for use in humans. First generation vaccines were largely successful at preventing severe disease and hospitalization caused by earlier variants of concern, including Beta and Delta. However, efficacy has waned with the emergence of the Omicron variant [10,11,44]. All currently approved vaccines use the original Wuhan sequence, the majority of which use the Spike as the sole antigen. Regularly updating and readministering vaccines using the current platforms as new variants continue to emerge will be unsustainable in the long term. New strategies that can induce a more broadly protective response need to be explored.

Here, we present preclinical immunogenicity and efficacy studies of VLP-based vaccines against SARS-CoV-2. The VLP platform provides significant advantages over currently approved vaccines. First, VLPs are structural mimics of viruses that are rendered non-infectious due to a lack of genetic material, rather than through the use of chemical inactivation processes used in the manufacturing of inactivated virus vaccines, which can reduce immunogenicity [26,27,28]. Additionally, coronavirus VLPs contain three additional antigens relative to the Spike-only vaccines, as all four structural proteins can be incorporated. Furthermore, there is no potential for pre-existing immunity against the vector with VLP-based vaccines, which can be an issue with adenovirus-vectored vaccines [46]. Finally, VLPs are known to be both safe and effective in humans in preventing other diseases, such as HPV and HBV [16,17,18,19,20].

In this study, we demonstrate that SARS-CoV-2 VLPs can be formed by expressing the structural proteins in a mammalian expression system via transfection, though a stable producer cell line will need to be developed to ensure scalability and lot-to-lot consistency. Purified VLPs showed a size and morphology comparable to the native virus, and contained three of the main structural proteins, S, M, and N. We were unable to detect E in Western blots of VLP samples, likely due to the low abundance of E relative to other structural proteins in fully formed coronavirus particles [41]. However, formation of coronavirus VLPs is dependent upon the expression of E [23]. Previous studies have shown that E is an important driver of membrane curvature, and mutations in E lead to an irregular morphology of assembled particles [47,48]. Given the necessity of E for VLP formation, and that the size and morphology of our VLPs were comparable to the native virus, we are confident that E was incorporated into the VLPs.

We investigated the effectiveness of formulating our VLPs in combination with a variety of adjuvants, including alum and a squalene-based oil-in-water emulsion analogous to MF59 (AddaVax), in comparison to non-adjuvanted VLPs. Immunization with the monovalent Beta VLP vaccine elicited high titers of Spike-specific binding- and neutralizing-antibodies in a mouse model. VLP vaccination in mice induced antibodies capable of neutralizing both the vaccine-homologous Beta variant and heterologous Delta variant, regardless of adjuvant formulation. IgG subclass analysis revealed an expected Th2 bias for the alum group. Strong Th2 responses have been associated with severe disease and poor prognosis [49,50]. In contrast, AddaVax elicited a more balanced Th1/Th2 response, which is important for limiting disease severity, especially in high-risk groups such as patients with asthma [51]. Interestingly, despite the heavily Th2-biased response elicited by the alum-adjuvanted VLPs, the AddaVax group elicited the highest titers of both total IgG and neutralizing antibodies. Additionally, MF59, the pharmaceutical equivalent of AddaVax, has been licensed for use in humans and is safe and well tolerated even in more sensitive populations [52,53]. Therefore, AddaVax was used in further studies of our VLP platform against SARS-CoV-2.

To assess the protective efficacy of the VLP platform, a viral challenge study was performed in Golden Syrian hamsters. The hamster model has been widely used in COVID-19 studies due to the high binding affinity of hamster ACE2 to the SARS-CoV-2 S protein [42,43], in addition to the development of clinical signs and lung pathology comparable to that observed in humans [42]. Hamsters were immunized with a bivalent VLP vaccine, composed of a combination of monovalent VLPs produced using either the Beta or Delta variant Spike protein, designed to induce a wider range of protection. Beta and Delta were selected given the significance of their antigenic distance [29,30], as convalescent sera from patients infected with Beta is typically poor at neutralizing Delta, and vice versa [29,31]. Additionally, Beta and Delta were the predominant circulating variants at the time the study was conducted. Humoral immunity was assessed in hamsters as well, yielding results comparable to those seen in mice, with immunized hamsters exhibiting robust production of S-specific binding- and neutralizing-antibodies. Furthermore, we also showed that immunization with our bivalent VLP vaccine induces cross-neutralizing antibodies against the Omicron BA.1 variant, though further study will be needed to understand the observed sex-based differences in cross-neutralizing activity.

After collection of sera, hamsters were challenged with SARS-CoV-2, and viral load was monitored over time in the oropharyngeal passages as a measure of viral shedding. By day 3 post challenge, all animals showed low or undetectable levels of virus in the oropharynx, even in control groups. This reduction in shedding occurred earlier in immunized hamsters than in control animals, suggesting that VLP vaccination could be beneficial in reducing transmission by shortening the duration of viral shedding. However, further studies are needed to determine the exact effect of vaccination with our VLP vaccines on transmission, as viral shedding was not prevented entirely. Vaccinated hamsters also showed significantly reduced viral load and replication in the lungs and nasal turbinates, areas which tend to show the highest viral titers across a wide range of sampled tissues in unvaccinated hamsters [42]. The most pronounced reduction in viral load was observed in the lungs. A lesser degree of protection was observed in the nasal turbinates, though hamsters are typically slow to clear infectious virus and viral RNA in the turbinates [42,54]. Although reports vary [55,56], lower viral titers are typically associated with decreased disease severity [57,58,59], indicating that VLP vaccination likely prevents severe disease.

Reduced viral load and replication in the lungs of vaccinated hamsters translated to significant protection against severe lung pathology when challenged with either the Beta or Delta variant. While VLP-immunized animals challenged with Beta showed minimal tissue damage, those challenged with Delta showed signs of moderate lung disease. These differences observed between challenge variants were expected, given that Delta is known to cause more severe disease than Beta in humans [60]. We found this disparity to be true in control hamsters as well, as Delta caused more severe pathology than Beta in unvaccinated animals. Importantly, while histopathological scores increased from day 3 to day 7 post-challenge in unvaccinated hamsters, VLP-immunized animals showed a decrease in scores between these timepoints, indicating a more rapid clearance of the infection and recovery.

Both the low- and high-dose bivalent VLP vaccine groups performed well overall, with both doses performing significantly better than control groups in several metrics. However, dose-dependent effects were observed, as the high dose group outperformed the low dose group in general. Against Delta, the high dose group showed significantly greater neutralizing activity, and significantly lower viral titers and viral RNA in the tissues as compared to the low dose group. Lower disease severity and tissue damage were also observed in the high dose group, though the differences did not reach the threshold for statistical significance. Against Beta, dose-dependent effects were observed for some metrics, such as neutralizing activity and viral swab titers. In contrast, tissue titers and histopathological scores fell near or below the lower limit of detection in both dosage groups, rendering any potential dose-dependent effects indiscernible. Further dose escalation studies are needed to determine the appropriate dose that yields protection whilst conserving antigen. This study should also be used to optimize the ratios of strain-specific monovalent VLPs combined in polyvalent vaccine formulations.

In summary, we demonstrate that the SARS-CoV-2 virus-like particles present an attractive platform for the development of the next generation vaccines against COVID-19, capable of inducing a potent humoral immune response and providing protection against severe disease.

## 5. Patents

M.D.R., K.W., R.M., H.M.M., M.P., K.G., L.G. and J.M.G. are current or former employees of TechnoVax, Inc., which has submitted a patent application (63/066,617) related to the methods of production and applications of SARS-CoV-2 VLPs.

## Data Availability

All data are available from the corresponding author upon request.

## Supplementary Materials

The following supporting information can be downloaded at: https://www.mdpi.com/article/10.3390/vaccines10121997/s1, Figure S1: Plasmid schematic; Figure S2: Full images of blots shown in Figure 1d–f.
