# Supplementary figures and images for: Immunogenicity and Efficacy of Monovalent and Bivalent Formulations of a Virus-Like Particle Vaccine against SARS-CoV-2
Source: Vaccines (Basel). 2022 Nov 24;10(12):1997. doi: 10.3390/vaccines10121997 (PMC9782034; doi:10.3390/vaccines10121997)

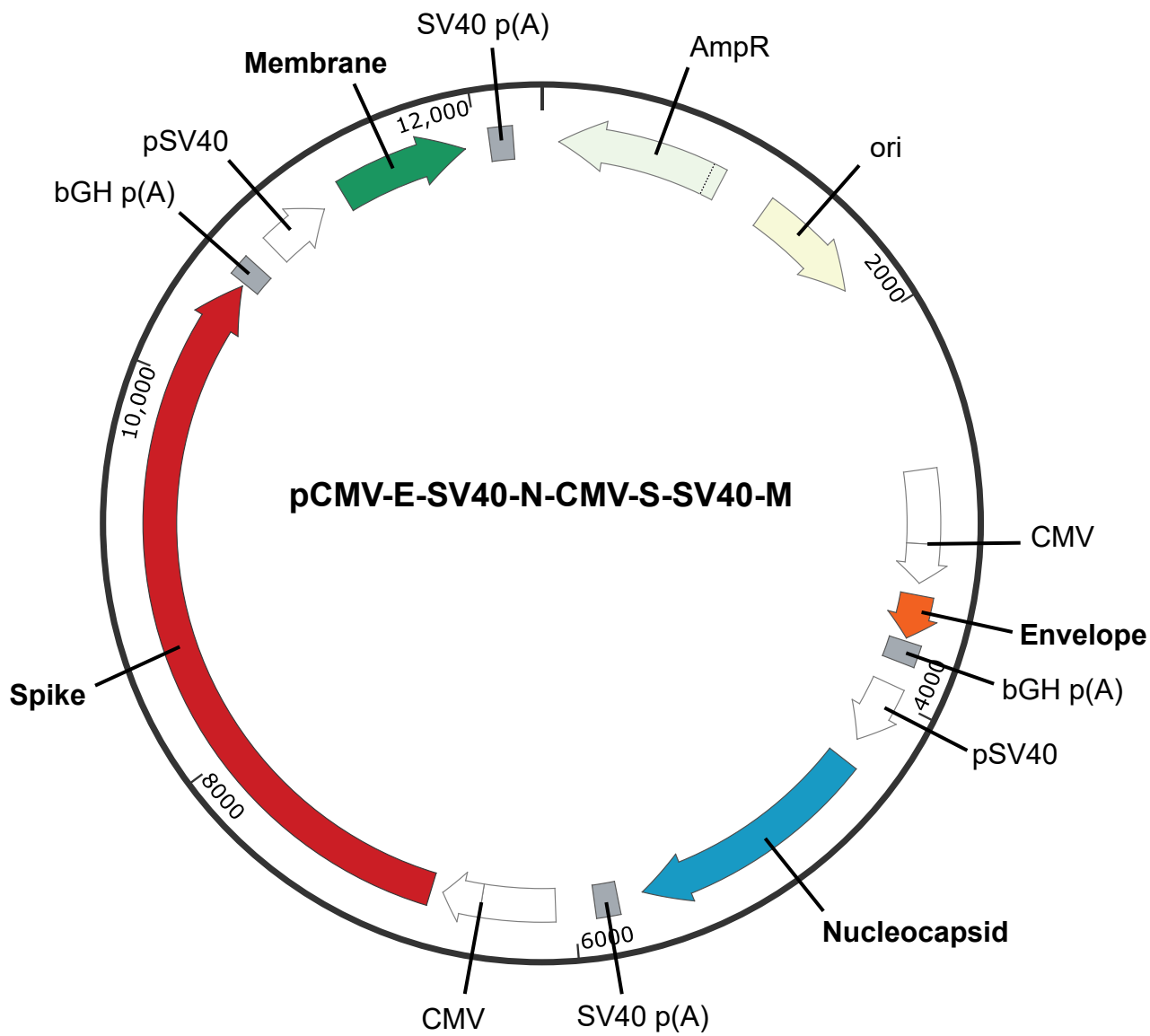

Supplement: Supplementary file 1 [file vaccines-10-01997-s001.zip › Figure S1.pdf]

**a**

**Spike**

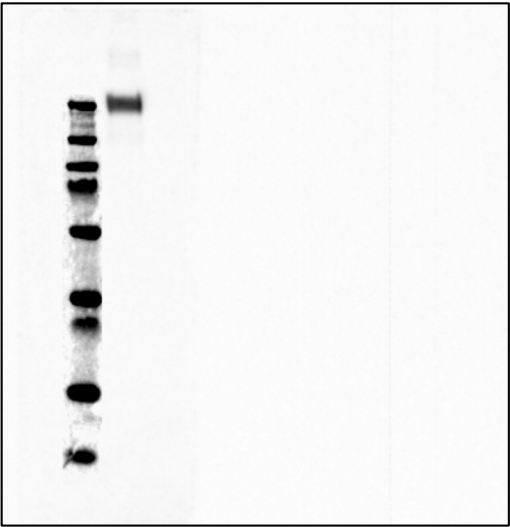

**b**

**Membrane**

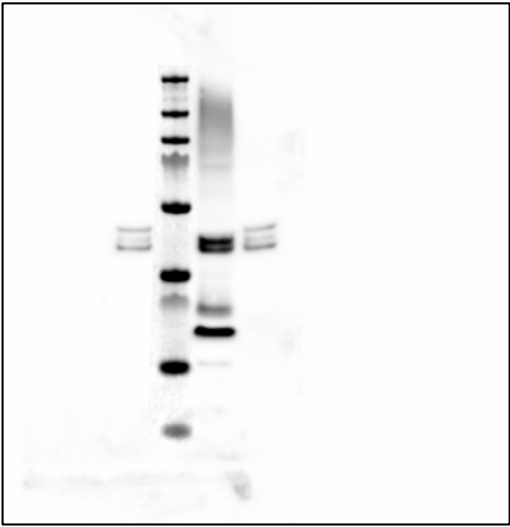

**c**

**Nucleocapsid**

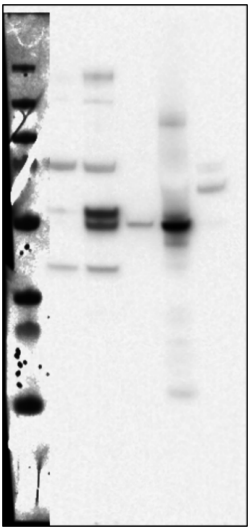

Supplement: Supplementary file 1 [file vaccines-10-01997-s001.zip › Figure S2_Revised.pdf]
